# Standardized mortality ratios between street-connected young people and the general age-equivalent population in an urban setting in Kenya from 2010 to 2015

**DOI:** 10.1080/16549716.2020.1802097

**Published:** 2020-08-21

**Authors:** Mia Kibel, James Pierzchalski, Lauren Gorfinkel, Lonnie Embleton, David Ayuku, Robert Hogg, Paula Braitstein

**Affiliations:** a Dalla Lana School of Public Health, Division of Epidemiology, University of Toronto, Toronto, ON, Canada; b Department of Epidemiology and Population Health, BC Centre for Excellence in HIV/AIDS, Vancouver, BC, Canada; c Faculty of Health Sciences, Simon Fraser University, Burnaby, BC, Canada; d Mailman School of Public Health, Columbia University, New York, NY, USA; e Institute of Medical Science, University of Toronto, Toronto, Canada; f College of Health Sciences, School of Medicine, Department of Behavioral Sciences, Moi University, Eldoret, Kenya; g College of Health Sciences, School of Medicine, Department of Medicine, Moi University, Eldoret, Kenya

**Keywords:** Homeless youth, street youth, mortality, Kenya, Africa, sub-Saharan

## Abstract

There are currently no published estimates of mortality rates among street-connected young people in Kenya. In this short report, we estimate mortality rates among street-connected young people in an urban setting in Kenya and calculate standardized mortality ratios to assess excess mortality among street-connected young people compared to the general population of Kenyan adolescents. We collected data on deaths among street-connected young people aged 0–29 between 2010 and 2015. We calculated sex-stratified standardized mortality ratios for street-connected young people aged 0–19 and 20–29 from 2010 to 2015, using publicly available Kenya population data as reference. We found that between 2010 and 2015, there were 69 deaths among street-connected young people aged 0 to 29 years in 2013 was 1,248: 341 females (27%) and 907 males (73%). The standardized mortality ratios among street-connected females aged 0–19 and 20–29 years were 2.79 (95% CI 1.44–4.88) and 7.55 (95% CI 3.77–13.51), respectively; standardized mortality ratios among street-connected males aged 0–19 and 20–29 years were 0.71 (95% CI 0.32–1.35) and 5.48 (95% CI 3.86–7.55), respectively. In conclusion, we found that mortality among street-connected young people in an urban setting in Kenya is elevated compared to the general population of Kenyan young people. States should act urgently and take responsibility for protecting street-connected young people’s human rights by scaling up programs to prevent morbidity and death associated with youth street involvement.

## Background

A recent publication described a high number of deaths among street-connected young people (SCY) in an urban setting in Kenya, mostly from HIV and violence [1], and another described an HIV prevalence of 4.1% in the same population [2]. SCY, broadly defined as young people who live and work on the streets or for whom the streets are a central part of their identity, experience a high burden of morbidity related to infectious diseases, mental health, substance use, and sexual health [3]. Primary causes of homelessness among young people are poverty, family conflict, and child abuse/neglect [4]. UNICEF estimates that there are tens to hundreds of millions of homeless or street-connected young people globally [5].

Despite evidence suggesting high mortality, there are currently no published estimates of mortality rates in populations of SCY in Kenya, or other countries in sub-Saharan Africa (SSA). The absence of data limits our ability to understand the burden of preventable death among SCY and develop evidence-based policies to reduce morbidity and mortality. The aim of this study is to estimate mortality rates among SCY in an urban setting in Kenya and calculate standardized mortality ratios to assess excess mortality among SCY compared to the general Kenyan population of the same age.

## Methods

### Study setting

Eldoret, 2019 population 475,716 [6], is home to Moi University, Moi Teaching and Referral Hospital, and the Academic Model Providing Access to Healthcare (AMPATH), which currently provides HIV care and other healthcare to over 80,000 people across Western Kenya.

### Study population

SCY were defined as individuals aged 0–29 years who either (a) spent both days and nights on the streets and had limited/no contact with caregivers, or (b) spent some/most of their time on the street and returned to caregivers at night [7]. The United Nations defines ‘youth’ as people aged 15–24 years [5]; however, we included individuals aged 0–29 years due to the difficulty confirming age in this population and setting.

### Study design

Data on the size of the total population of SCY in Eldoret were collected in 2016 via a point-in-time (PIT) count. PIT counts are a census-type approach to counting homeless individuals at a given time point. The methods are described in Braitstein et al. 2019. In brief, after extensive community consultation, four teams were stationed at sites where SCY are known to gather. Consenting SCY provided information including age, sex, and where they slept at night. Participant’s fingerprints were digitally captured to prevent double-counting, and participants were connected to HIV testing and counseling [2].

Data on deaths among SCY in Eldoret were collected between 2010 and 2015. Methods are described in Embleton et al. 2018 [1]. Briefly, between 2010 and 2013, a community advocate with in-depth knowledge of Eldoret’s community of SCY recorded deaths among SCY, including age, sex, and cause of death. Records were cross-referenced with Eldoret mortuary data to ensure completeness. From 2013 to 2015, deaths among SCY were recorded via standardized forms when reported to a community advocate or identified at the Eldoret mortuary. Completeness of the data may be limited given that some deaths among SCY may not have been reported, and some young persons may not have been identified as SCY at the mortuary. However, given the advocate’s strong connection to the community of SCY, these records are likely relatively complete. Only deaths identified between 2010 and 2015 among SCY aged 0–29 years with known sex were included. Ethics approval was received from Moi University and the University of Toronto Research Ethics Boards.

Data on the 2013 population and the 2010 to 2015 mortality rate among Kenyan individuals aged 0–29 years were obtained via World Population Prospects (WPP) 2017, a free online source for the United Nation’s global population estimates and projections. WPP provides annual estimates for Kenya’s population and 5-year period estimates for deaths by age category (0–4, 5–9, 10–14, 15–19, 20–24, and 25–29 years). WPP estimates are based on analyses of all available information and relevant historical demographic trends, including national census data [8].

### Statistical methods

Age- and sex-stratified standardized mortality ratios (SMRs) were calculated by dividing the observed by expected number of deaths in the population of SCY from 2010 to 2015. The expected number of deaths were calculated by multiplying the Kenyan population death rate from 2010 to 2015 by the total age-equivalent population of SCY in 2013. The total population of SCY in Eldoret in 2013 was back-calculated by applying the rate of change in the Kenyan <30 population from 2013 to 2016 to the 2016 SCY PIT count data, since only one PIT count has ever been conducted among SCY in Eldoret. Data were stratified by sex and age categories 0–19 and 20–29 years. Age groups from 0–19 and 20– 29 were combined to increase the validity of mortality rate estimates. Ninety-five per cent confidence intervals (CI) were calculated in R version 3.6.1 using the exact two-sided Poisson test (Package exactci version 1.1–3) [9].

## Results

Between 2010 and 2015, there were 69 deaths among SCY aged 0–29 years: 23 females (33%) and 46 males (67%). The total 2016 population of SCY <30 years of age was 1,327: 360 females (27%) and 967 males (73%). The estimated population of SCY <30 years of age in 2013 was 1,248: 341 females (27%) and 907 males (73%). Population and deaths by age are presented in Table 1.

**Table 1.** Population and number of deaths among street-connected young people in Eldoret, Kenya between 2010 and 2015.

	SCY population 2016	SCY estimated population 2013	SCY deaths 2010–2015	SCY mortality rate per 1000, 2010–2015	Kenya mortality rate (aged 0–19 and 20–29) per 1000, 2010–2015
Males, by age					
0–4	79	78	2		
5–9	65	61	1		
10–14	157	146	3		
15–19	241	219	3		
0–19	**542**	**504**	**9**	**17.9**	**25.2**
20–24	220	208	21		
25–29	205	195	16		
20–29	**425**	**403**	**37**	**91.8**	**16.8**
Females, by age					
0–4	66	65	7		
5–9	47	44	1		
10–14	62	58	1		
15–19	52	47	3		
0–19	**227**	**214**	**12**	**56.0**	**20.0**
20–24	83	79	6		
25–29	50	48	5		
20–29	**133**	**126**	**11**	**87.2**	**11.6**

**Figure 1.**
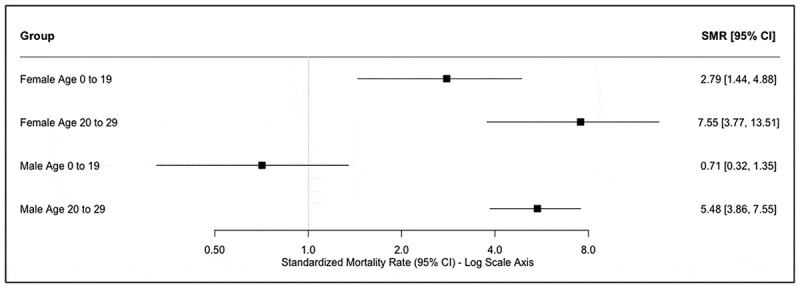
SMR of SCY in Eldoret, Kenya compared to general population of Kenyan young people between 2010 and 2015.

The SMR among female SCY aged 0-19 and 20-29 years were 2.79 (95% CI 1.44-4.88) and 7.55 (95% CI 3.77-13.51), respectively. The SMR among male SCY aged 0-19 and 20-29 years were 0.71 (95% CI 0.32-1.35) and 5.48 (95% CI 3.86-7.55), respectively (Figure 1).

## Discussion

Our findings suggest that there is an elevated burden of mortality among SCY in Kenya compared to the general population of Kenyan young people.

This work, to our knowledge, is the first to quantify the burden of excess mortality in SCY in Kenya, or any country in SSA. Our findings are broadly consistent with studies in North America, Europe, and Australia which have reported mortality rates in SCY between 2.1 and 37.3 times greater than mortality rates in the local general population of the same age. These analyses also found that SMRs were greater in female SCY than in male SCY, suggesting women and girls living on the street are especially vulnerable to harm [10]. In this study, male SCY aged 0–19 years had a lower mortality rate than the age-equivalent general population; however, this result was not statistically significant. More research is needed to understand mortality risk among young male SCY. Possible protective factors for young male SCY include stronger social networks and more diverse strategies for earning money compared to female SCY [11], and self-selection among boys who come to the street to escape poverty and family conflict [4].

Embleton et al.’s report on causes of death among SCY in Eldoret demonstrated that the majority of deaths were related to HIV/AIDS among female SCY, and violence or accidents among male SCY [1]. Drivers of SCY’s mortality in this urban setting in Kenya likey differ from high-income countries, where SCY deaths are more often related to suicide and substance use [10,12].

Our findings are limited by small sample size, potentially incomplete capture of deaths among SCY, and only one measurement of the population of SCY in Eldoret. Furthermore, Eldoret is home to a large hospital and health program (AMPATH) where SCY may be able to access HIV care and supports not available in all other settings. However, our results should signal to policy makers and human rights advocates that SCY in Eldoret are dying of preventable causes at rates well above other young people, and that additional studies of SCY’s mortality and related risk factors across Kenya and SSA are urgently needed. Poverty is a major cause of youth street involvement [4], and thus climate change, COVID-19, the ongoing HIV epidemic, and other drivers of economic instability may contribute to increased youth homelessness and mortality in the future. States should act immediately to protect SCY’s Right to Life and Right to Survival and Development, as outlined in the United Nations Convention on Rights of the Child [7], by scaling up programs to prevent morbidity and death associated with street involvement.

## Paper context

Our work shows a high mortality rate and excess mortality among street-connected young people in an urban setting in Kenya, and is the first published estimate of mortality among street-connected young people in Kenya. States should act urgently on their responsibility to protect street-connected young people’s human rights by scaling up programs to prevent morbidity and death associated with youth street involvement.

## References

[1] Embleton L, Ayuku D, Makori D, et al. Causes of death among street-connected children and youth in Eldoret, Kenya. BMC Int Health Hum Rights. 2018;18:19. 29764412 10.1186/s12914-018-0160-8 PMC5952842

[2] Braitstein P, Ayuku D, DeLong A, et al. HIV prevalence in young people and children living on the streets, Kenya. Bull World Health Organ. 2019;97:33–4. Epub 2019 109. PubMed PMID: 30618463; PubMed Central PMCID: PMCPMC6307507. 30618463 10.2471/BLT.18.210211 PMC6307507

[3] Woan J, Lin J, Auerswald C. The health status of street children and youth in low-and middle-income countries: a systematic review of the literature. J Adolesc Health. 2013;53:314–21. e12. 23706729 10.1016/j.jadohealth.2013.03.013

[4] Embleton L, Lee H, Gunn J, et al. Causes of child and youth homelessness in developed and developing countries: a systematic review and meta-analysis. JAMA Pediatr. 2016;170:435–444. 27043891 10.1001/jamapediatrics.2016.0156 PMC5497301

[5] Unicef. The state of the world’s children 2012: children in an urban world. 2012. https://www.unicef.org/sowc/index_61804.html.

[6] Kenya National Bureau of Statistics. 2019 Kenya population and housing census, Volume II: distribution of population by administrative units. Nairobi, Kenya: Kenya National Bureau of Statistics; 2019.

[7] United Nations Committee on the Rights of the Child. General comment No. 21 (2017) on children in street situations. Geneva, Switzerland: United Nations Committee on the Rights of the Child; 2017.

[8] United Nations Population Division. 2017 revision of world population prospects. 2017. https://population.un.org/wpp/.

[9] Fay MP Two-sided exact tests and matching confidence intervals for discrete data. R Journal. 2010;2:53–58.

[10] Auerswald CL, Lin JS, Parriott A. Six-year mortality in a street-recruited cohort of homeless youth in San Francisco, California. PeerJ. 2016 Epub 2016 427;4:e1909. PubMed PMID: 27114873; PubMed Central PMCID: PMCPMC4841235. 27114873 10.7717/peerj.1909 PMC4841235

[11] Sorber R, Winston S, Koech J, et al. Social and economic characteristics of street youth by gender and level of street involvement in Eldoret, Kenya. PLoS One. 2014;9:5. 10.1371/journal.pone.0097587 PMC4020866 24827584

[12] É R, Haley N, Boudreau J-F, et al. The challenge of understanding mortality changes among street youth. J Urban Health. 2010;87:95–101. PubMed PMID: PMC2821604. 20039140 10.1007/s11524-009-9397-9 PMC2821604

